# Dietary nitrate supplementation reduces performance decline during repeated sprint exercise independent of sex

**DOI:** 10.5114/biolsport.2026.162401

**Published:** 2026-06-08

**Authors:** Yadan He, Xiaochuan Ge, Chunxue Tang, Yanqiu Wang, Haizheng Huang, Bin Leng, Chuan Zhang

**Affiliations:** 1 School of Physical Education and Sports, Central China Normal University, Wuhan, China; 2 School of Kinesiology, Beijing Sport University, Beijing, China

**Keywords:** Peak performance, Transcranial magnetic stimulation, Near-infrared spectroscopy, Dietary nitrate, Repeated sprints

## Abstract

Dietary nitrate supplementation has been shown to improve endurance exercise performance, but its effects on repeated anaerobic sprints and potential sex-based differences remain underexplored. This study examined whether acute dietary nitrate supplementation reduces performance decline during repeated Wingate sprints and whether responses differ between sexes. The secondary aim was to investigate the potential central and peripheral mechanisms. Using a randomized crossover design, 19 males and 17 females (all recreational athletes) consumed dietary nitrate supplementation in the form of nitrate-rich beetroot juice (~13 mmol NO_3_^−^) or placebo 2.5 hours prior to completing four 30-second maximal cycling sprints. The primary outcomes included power output and total work changes from the first to the last sprint. To investigate the potential mechanisms, corticospinal excitability and muscle microvascular function were also measured prior to sprints. No sex by supplement interactions were observed (all p > 0.05) for any of the measured parameters. Dietary nitrate supplementation significantly attenuated the decline in performance across repeated sprints, with large main effects on peak power, mean power, power decline, and total work (all p < 0.05). However, dietary nitrate supplementation did not affect corticospinal excitability or muscle microvascular reactivity (all p > 0.05). Acute dietary nitrate ingestion effectively mitigates fatigue development during repeated anaerobic efforts; however, no sex differences were detected. The observed performance benefits occurred without detectable changes in resting measures of corticospinal excitability or microvascular reactivity.

## INTRODUCTION

Beetroot juice (BJ) has become a widely used ergogenic aid due to its high concentration of dietary inorganic nitrate (NO_3_^−^). The ingested NO_3_^−^ is reduced to nitrite (NO_2_^−^) and subsequently forming nitric oxide (NO), which affects a variety of physiological mechanisms [1]. As a potent vasodilator, NO can lead to increased blood vessel diameters, thereby enhancing blood flow to the downstream tissues [2]. Additionally, NO also plays an important role in metabolic regulation. It has been shown that NO improves mitochondrial efficiency by enhancing oxidative phosphorylation and reducing proton leak, thereby allowing mitochondria to generate more ATP per unit of oxygen consumed [3]. Furthermore, recent studies have shown that NO may be able to modulate maximal muscle contractility through effects on Ca^2+^ release and sarcolemmal excitability [4]. These combined effects make BJ a potentially ideal supplement for those aiming at improving sports performance.

The ergogenic effects of dietary NO_3_^−^ on performance have been most consistently reported in aerobic and submaximal exercises. Many studies have shown that acute or chronic NO_3_^−^ supplementation could reduce the oxygen cost during exercise, delay fatigue, improve time-to-exhaustion and time-trial performance in endurance exercises [5, 6]. On the other hand, the effects of dietary NO_3_^−^ on high-intensity, predominantly anaerobic performance remain less clear. Studies that examined single sprint performance have yielded conflicting results, with some suggesting noticeable improvements [7], while others reported negligible changes in outcomes [8]. It’s possible that the effects of dietary NO_3_^−^ on short, single bout of anaerobic exercise are variable and dependent on many other factors, such as exercise protocol and training status [9].

The inconsistent findings from single-sprint studies have also prompted the investigation to repeated-sprint protocols, which may be more suitable for detecting nitrate’s ergogenic potential. During sprints, substantial metabolic stress is induced, including phosphocreatine depletion, decline in pH, and accumulation of metabolic byproducts such as lactate and hydrogen ions [10]. During recovery between two consecutive sprints, the ability to rapidly restore phosphocreatine stores and clear metabolic by-products is influenced by muscle perfusion, a process that dietary NO_3_^−^ may enhance through increased NO availability. Some studies have found that dietary NO_3_^−^ supplementation could help maintain power output or speed during repeated cycling or running [11–13]. However, research in this area is still limited, the findings to date remain inconsistent [14, 15], and the underlying mechanisms remain to be elucidated [16]. A recent meta-analysis investigating the effects of dietary nitrate on repeated sprint performance revealed conflicting and inconsistent findings, with a small but significant effect for mean power but not for peak power or total work done, and large heterogeneities across studies [17]. Therefore, while the ergogenic effects of dietary nitrate on high-intensity and repeated-sprint exercise have been studied, the results are not unequivocal and still warrant further investigation. Besides peripheral factors, repeated-sprint fatigue is also associated with reductions in central motor drive [18]. Given that recent studies found increased blood flow to the brain following NO_3_^−^ supplementation [19, 20], it’s possible that NO_3_^−^ may also exert its effects by modulating the excitability of the central nervous system, thus maintaining corticospinal output during high-intensity efforts, however this mechanism is yet to be investigated. Taken together, while performance benefits are documented in some studies, explanatory mechanisms for fatigue attenuation across repeated bouts remain unresolved and need to be further investigated.

Another aspect worth exploring is the potential sex-dependent response to nitrate supplementation. Studies have pointed out that females are substantially underrepresented in sport-related studies [21]. A recent umbrella review has highlighted the substantial underrepresentation of females in dietary nitrate research. Among the 11 reviews reporting sex ratios, women comprised just 7–37% of pooled samples [5]. Additionally, only 14% (56/410) of participants in the previously mentioned repeated-sprint meta-analysis were females [17]. These reviews underscored that such underrepresentation limits the ability to determine whether ergogenic responses are consistent across sexes and constrains the external validity of current recommendations. Biological sex could influence nitrate metabolism and hormone levels. For example, estrogen has been shown to upregulate NO synthase activity and alter vascular responsiveness [22]. However, to our knowledge, studies specifically examining sex differences in nitrate’s ergogenic effects during repeated anaerobic exercise have not been reported. Given the different responses seen in previous studies focusing on other exercise types, such as endurance exercises [23], it is plausible that males and females may also exhibit distinct physiological responses to nitrate during repeated anaerobic efforts; however, this has yet to be tested. To address the gaps in the literature, the primary purpose of our study was to determine whether dietary nitrate supplementation in the form of BJ reduces performance decline during repeated Wingate sprints and whether responses differ between sexes. The secondary purpose of this study was to investigate the potential central and peripheral mechanisms.

## MATERIALS AND METHODS

### Participants

This study recruited young healthy recreational males (n = 19) and females (n = 17) athletes. The inclusion criteria were 1) physically active, defined as regularly practicing physical exercise for at least 5 times per week, and lasts at least 1 hour per session, 2) generally healthy without any known cardiovascular or musculoskeletal diseases, and free from injury to the lower extremities for the past year, 3) having regular menstruation for female participants, and 4) not currently taking any beetroot-based supplementations.

### Ethics

This study was approved by the Institutional Review Board at our University, and all study procedures were carried out according to the Declaration of Helsinki. All participants signed a written consent form prior to any data collection.

### Study Design

This is a randomized cross-over trial. The participant first reported to our laboratory to sign the written consent form, and was also given the chance to familiarize with the testing procedures. On the first testing day, the participant was given an opaque juice bag, which contains either 140 ml of concentrated NO_3_^−^ rich BJ (13 mmol of NO_3_^−^; Beet It, James White Drinks Ltd., Ipswich, UK) [24] or equivalent amount of placebo (PL, low-calorie black-currant juice with negligible NO_3_^−^ content) [25, 26]. The BJ and PL supplementation were slightly different in terms of taste, however, as none of our study participants was familiar with the taste of BJ, they could not tell the exact nature of the supplement they received. Participants consumed the assigned beverage in the juice bag 2.5 hours prior to the testing [27], which is required to be at least 2 hours after the last meal. This procedure was repeated 2–7 days later at a similar time, except those who received BJ last time received PL, and vice versa. For each testing session, participants completed a corticospinal excitability test, a microvascular function assessment, and a repeated Wingate test.

**FIG. 1:**
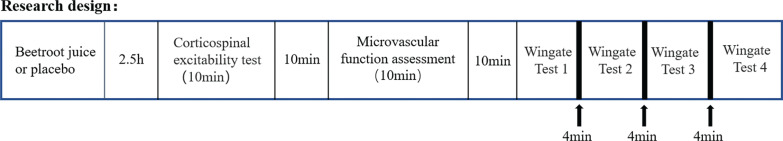
Schematic of the research design.

Female participants were tested during the late follicular phase of the menstrual cycle (verified by calendar tracking based on selfreported date of last menstruation and typical cycle length) to avoid the potential influence of hormonal fluctuation. Throughout the study period, the participants were given a list of nitrate-rich foods (e.g., beetroot, spinach, broccoli) and were instructed to avoid their consumption. The participants were asked to refrain from performing strenuous exercise and drinking alcohol 24 hours prior to each testing session. They were also asked to refrain from drinking any caffeinated drinks on the days of testing. A schematic for the research design is shown in Figure 1.

### Corticospinal Excitability

Transcranial magnetic stimulation (TMS) was administered to the left primary motor cortex (M1) using a figure-eight coil (loop diameter: 9.5 cm) connected to a Magstim 200 stimulator (Magstim, Whitland, Dyfed, UK). For paired-pulse protocols, a second Magstim 200 stimulator was employed. Coil orientation was maintained with the handle pointing posterolaterally at 30–45° relative to the midsagittal plane, thereby inducing a posterior-anterior current flow approximately perpendicular to the central sulcus. Electromyographic (EMG) activity of the right first dorsal interosseous (FDI) muscle was recorded using 9-mm Ag-AgCl electrodes. The active electrode was positioned over the belly of the FDI muscle, and the reference electrode was placed over the proximal interphalangeal joint of the right index finger. The ground electrode was attached to the radial aspect of the right forearm. The EMG signals were amplified with a gain of 1000 to ensure adequate motor evoked potential (MEP) signal amplification, and the signal was bandpass-filtered between 20 and 2500 Hz.

The motor hotspot was identified as the scalp position eliciting maximal peak-to-peak MEP in the FDI muscle. This position was marked using a permanent marker for consistent placement for the next testing session. Individualized stimulation intensities were determined prior to the experimental session. Resting motor threshold (RMT) was defined as the lowest stimulator output producing MEPs exceeding 50 μV in at least five of ten consecutive trials during complete muscle relaxation. One mV MEP was identified as the lowest stimulator output required to generate MEPs surpassing 1 mV in at least five of ten trials under resting conditions. Both RMT and 1 mV MEP values represent the percentage of maximum stimulator output.

### Microvascular Function Assessment

Skeletal muscle microvascular function was assessed using nearinfrared spectroscopy (NIRS; 58 × 28 × 6 mm, PortaLite, Artinis Medical Systems B.V., Einsteinweg, The Netherlands) coupled with the post-occlusive reactive hyperemia technique. While lying supine, a continuous wavelength NIRS probe was placed on the right vastus lateralis (VL) at one-third distance between the patella and the anterior superior iliac spine (closer to the patella), and was marked using a permanent marker to ensure consistent placement across all testing sessions. A 11-cm tourniquet connected to a custom-built rapid cuff inflation device was placed as proximally as possible on the thigh to provide full vascular occlusion.

After lying on the bed for at least 10 minutes, the NIRS device was activated to capture 1–2 minutes of baseline data. Subsequently, the tourniquet was rapidly inflated to ~250 mm Hg to achieve complete occlusion of downstream blood flow to the VL. This ischemic state was maintained for 5 minutes, after which the tourniquet was rapidly deflated to restore blood flow. NIRS monitoring was continuous throughout the entire process, with an additional 3 minutes post-deflation to capture the full hyperemic response.

The obtained NIRS data were processed using a custom-written Matlab (version 2020b, The Mathworks, Natick, MA, USA) script. The NIRS device exploits the light absorption characteristic differences between oxygenated and deoxygenated hemoglobin, and thereby provides relative changes of their concentrations. The device also provides tissue saturation index (TSI), which is an indicator commonly used to represent muscle oxygenation. Key microvascular function parameters, including 1) the first 10 s of TSI recovery slope immediately following tourniquet release, TSI10, 2) the time it takes for the TSI signal to recover to half of the total recovery magnitude, TSI1/2, and 3) the total recovery magnitude, TSI_recovery_, were calculated as metrics for microvascular function [28–30].

### Repeated Wingate Tests

Participants performed four 30-second maximal cycling sprints on a mechanically braked cycle ergometer (Monark 894E, Sweden) against a resistance equivalent to 7.5% of their total body mass (kg). The resistance load coefficient was selected because it represents a widely used and standardized loading scheme in repeated Wingate protocols involving both males and females [31, 32]. This uniform relative load ensured comparable performance changes across sexes. The test started with a standard warm-up consisting of a 5-minute warm-up at a self-selected speed without resistance, and two 3–5 sec practice sprints. Afterwards, the participants started pedaling at a comfortable speed with no resistance, and after a 5-sec countdown, the resistance was applied, and the participants sprinted maximally for 30 s. A NIRS device was placed on the right VL on the same location as the microvascular function test, with the purpose of measuring muscle oxygenation changes.

This procedure was repeated three more times (without warmup for the follow-up tests). Between two consecutive tests, a 4-minute passive rest period was given to allow for recovery, during which the participants sat on the bike while no pedaling was allowed. Strong verbal encouragement was given by a research assistant throughout the entire test.

Power output was recorded using the Monark Anaerobic Test Software (version 3.3). For each sprint, the following variables were obtained: 1) peak power (PP), defined as the highest 5-second average power output, 2) mean power (MP), defined as the average power output across the entire 30 s trial, 3) power drop (PD), defined as the difference between the maximal and minimal power during each sprint, 4) total work performed throughout the 30 s (TW), 5) time-to-peak power (TTP), and 6) fatigue index (FI, percent decline from peak to minimal power in 5 s bins). Power-related variables (PP, MP and PD) were normalized to body mass. Muscle oxygen extraction was calculated as the difference between rest and the average of the last 5 s TSI values during the sprint (ΔTSI). To capture the potential nuance changes in these variables across the sprints, repeated Wingate test performance was quantified as the percent change in the above variables from sprint 1 to sprint 4 (PP_1–4_, MP_1–4_, PD_1–4_, TW_1–4_, TTP_1–4_ and FI_1–4_). This approach provides a single, interpretable index of performance decline and has been used in previous repeated-sprint studies [33, 34]. Muscle oxygen extraction change was calculated as the change in ΔTSI from sprint 1 to sprint 4 (ΔTSI_1–4_).

### Statistics

Sample size estimation was carried out using the G*Power software (G*Power 3.1, Heinrich-Heine-Universität Düsseldorf, Düsseldorf, Germany) [35]. Assuming an estimated moderate effect size of 0.25, an alpha level of 0.05, and power (1-β) of 0.8, it was estimated that 17 participants are required for each group. All statistical analyses were carried out using SPSS 27.0 (IBM Corp., Armonk, NY, USA). Two-way repeated-measures ANOVA was used with one betweengroups factor (sex) and one within-group factor (BJ or PL). Partial eta squared (partial η^2^) was calculated to indicate effect sizes of the results, with partial η^2^ < 0.06 indicating a small effect size, 0.06 ≤ partial η^2^ < 0.14 indicating a medium effect size, and partial η^2^ ≥ 0.14 indicating a large effect size. The alpha level was set at 0.05.

## RESULTS

A total of 19 male participants and 17 female participants volunteered for the study. The physical characteristics of the participants are summarized in Table 1.

**TABLE 1:** Physical characteristics for all participants.

	All (n = 36)	Male (n = 19)	Female (n = 17)	p-value
Age (years)	20.8 ± 1.6	20.8 ± 1.7	20.8 ± 1.4	0.883
Height (m)	1.7 ± 0.1	1.8 ± 0.1	1.7 ± 0.1	< 0.001
Weight (kg)	67.5 ± 9.8	72.9 ± 8.0	61.5 ± 8.1	< 0.001
BMI (kg/m^2^)	22.6 ± 1.9	23.2 ± 1.7	22.0 ± 2.0	0.065

P-values reflect comparisons between male and female participants only.

### Corticospinal Function (TMS)

The results of TMS revealed no significant effects of dietary nitrate supplementation on corticospinal or intracortical function. No significant sex × supplement interactions were present for either TMS measure (both p > 0.05). 1 mV MEP amplitude showed no supplement main effect (p = 0.217, partial η^2^ = 0.04). RMT also remained unchanged following dietary nitrate supplementation (p = 0.067, partial η^2^ = 0.10). Neither parameter showed a significant sex main effect (both p > 0.05, partial η^2^ < 0.014; Table 2).

**TABLE 2:** Results for corticospinal excitability test and muscle microvascular function test.

	Male (n = 19)		Female (n = 17)		p-value for supplementation	p-value for interaction	p-value for sex	partial η^2^ for sex

	BJ	PL	BJ	PL			
TSI_recovery_ (%)	33.4 ± 7.9	34.4 ± 7.5	16.8 ± 5.7	17.2 ± 5.3	0.522	0.789	< 0.001	0.682
TSI1/2 (s)	10.1 ± 1.7	9.2 ± 1.7	9.7 ± 3.6	9.2 ± 3.6	0.275	0.710	0.808	0.002
TSI10 (%/s)	1.7 ± 0.6	1.9 ± 0.6	0.8 ± 0.3	0.8 ± 0.3	0.132	0.276	< 0.001	0.584
1 mv MEP (%)	46.7 ± 8.4	43.8 ± 8.8	47.3 ± 8.9	46.8 ± 7.6	0.217	0.391	0.485	0.014
RMT (%)	18.0 ± 8.9	14.3 ± 7.8	16.4 ± 8.7	13.7 ± 7.4	0.067	0.769	0.600	0.008

BJ, beetroot juice consumption; PL, placebo consumption; TSI_recovery_, total recovery magnitude; TSI1/2, the time it takes for the TSI signal to recovery to half of the total recovery magnitude; TSI10, first 10 s of TSI recovery slope immediately following tourniquet release; 1 mv MEP, the lowest stimulator output required to generate motor evoked potentials surpassing 1 mV, values represent the percentage of maximum stimulator output; RMT, resting motor threshold, values represent the percentage of maximum stimulator output.

### Muscle Microvascular Reactivity and Oxygenation (NIRS)

NIRS measures of muscle microvascular function were unaffected by dietary nitrate supplementation. No significant sex × supplement interactions emerged for any NIRS parameter (all p > 0.05). Following tourniquet release, no significant within-subjects effects were observed for TSI_recovery_ (p = 0.522, partial η^2^ = 0.010). Similarly, TSI1/2 and TSI10 showed no supplement effects (p = 0.275 and p = 0.132, partial η^2^ = 0.035 and 0.065, respectively). Betweensubjects analyses revealed large sex differences in TSI10 and TSIr_ecovery_ (both p ≤ 0.001, partial η^2^ = 0.584 and 0.682, respectively), with males demonstrating higher values.

NIRS measured muscle oxygenation changes during repeated Wingate tests showed no interaction effect (p = 0.826, partial η^2^ = 0.001), nor was there a sex (p = 0.299, partial η^2^ = 0.032) or supplement (p = 0.483, partial η^2^ = 0.015) main effect. These findings indicate that acute dietary nitrate does not alter resting muscle microvascular reactivity or oxygenation dynamics during repeated anaerobic exercise.

### Repeated Wingate Anaerobic Performance

No significant sex by supplement interactions were observed for any Wingate variable (all p > 0.05), suggesting comparable ergogenic responses between males and females. On the other hand, acute dietary nitrate supplementation significantly attenuated repeated sprint performance decline compared to placebo. There was a large main effect of supplement for PP_1–4_ (p < 0.001, partial η^2^ = 0.307, MP_1–4_ (p < 0.001, partial η^2^ = 0.321) and TW_1–4_ (p < 0.001, partial η^2^ = 0.303). PD_1–4_ also demonstrated a significant supplement effect (p = 0.022, partial η^2^ = 0.146). On the other hand, no supplement main effect was detected for TTP_1–4_ (p = 0.095, partial η^2^ = 0.080) or FI_1–4_ (p = 0.175, partial η^2^ = 0.053). Between-subjects analyses revealed significant sex differences for PP_1–4_, MP_1–4_, TW_1–4_ (all p < 0.05, partial η^2^ = 0.193, 0.293 and 0.270, respectively), with males exhibiting higher values, indicating a higher rate of decline in performance (Table 3). However, this was not the case for PD_1–4_ (p = 0.110, partial η^2^ = 0.073), TTP_1–4_ (p = 0.197, partial η^2^ = 0.049) or FI_1–4_ (p = 0.734, partial η^2^ = 0.003).

**TABLE 3:** Performance variable changes in repeated Wingate tests. Peak Power, mean power and power drop are normalized values by dividing the raw values to body weight.

	BJ1	BJ2	BJ3	BJ4	PL1	PL2	PL3	PL4
**Males (n = 19)**								
Peak Power (W/kg)	10.4 ± 0.9	9.3 ± 1.1	7.9 ± 1.0	7.8 ± 0.9	11.0 ± 1.5	9.4 ± 1.3	7.8 ± 1.7	7.1 ± 1.7
Mean Power (W/kg)	7.8 ± 0.6	6.5 ± 0.5	5.4 ± 0.5	5.4 ± 0.6	8.0 ± 0.8	6.5 ± 0.7	5.4 ± 0.9	5.0 ± 0.9
Total Work (J)	16045 ± 1873	13350 ± 1763	11177 ± 1858	11052 ± 1831	16629 ± 2423	13300 ± 1869	10981 ± 1693	10259 ± 1880
Fatigue Index (%)	40.5 ± 10.1	42.8 ± 9.2	38.4 ± 12.0	42.3 ± 11.0	40.6 ± 8.6	44.6 ± 9.5	37.0 ± 13.5	41.3 ± 14.7
Time-to-Peak Power (s)	3.6 ± 2.0	3.1 ± 1.9	4.3 ± 2.6	5.5 ± 3.7	4.1 ± 1.9	2.7 ± 1.7	5.0 ± 4.5	8.1 ± 5.2
Power Drop (W/kg)	4.3 ± 1.3	3.9 ± 0.8	4.0 ± 1.0	4.0 ± 1.2	4.4 ± 1.0	4.2 ± 0.9	4.0 ± 1.1	3.8 ± 1.2

**Females (n = 17)**								
Peak Power (W/kg)	8.1 ± 1.3	7.3 ± 0.9	6.9 ± 0.9	6.6 ± 1.0	8.3 ± 1.3	7.1 ± 1.1	6.5 ± 1.0	6.2 ± 1.0
Mean Power (W/kg)	6.0 ± 0.8	5.3 ± 0.7	4.8 ± 0.6	4.7 ± 0.5	6.1 ± 0.9	5.1 ± 0.8	4.5 ± 0.7	4.4 ± 0.6
Total Work (J)	10588 ± 1660	9372 ± 1567	8291 ± 1413	8093 ± 1266	10716 ± 1749	8928 ± 1726	7878 ± 1514	7689 ± 1507
Fatigue Index (%)	34.6 ± 7.4	36.5 ± 7.0	38.7 ± 12.0	40.0 ± 12.8	36.7 ± 6.6	38.3 ± 7.6	39.5 ± 14.7	37.4 ± 14.3
Time-to-Peak Power (s)	3.4 ± 1.6	3.9 ± 2.7	3.9 ± 2.7	4.2 ± 2.6	4.0 ± 1.9	4.0 ± 2.5	4.0 ± 2.2	6.0 ± 6.6
Power Drop (W/kg)	5.9 ± 1.3	5.9 ± 1.0	4.7 ± 1.2	5.0 ± 1.3	6.9 ± 2.0	5.9 ± 1.3	5.0 ± 1.8	4.5 ± 1.8

BJ1-BJ4, Wingate test 1 to Wingate test 4 performance variables after consuming beetroot juice; PL1-PL4, Wingate test 1 to Wingate test 4 performance variables after consuming placebo.

## DISCUSSION

In this study, we found that acute dietary nitrate supplementation effectively attenuated performance decline during repeated Wingate sprints in both male and female recreational athletes, with large effect sizes for PP, MP, PD and total work. For the second purpose of the study, the absence of sex by supplement interactions indicates comparable ergogenic responses between sexes. However, the lack of detectable changes in corticospinal excitability or muscle microvascular reactivity suggests these peripheral and central pathways may not explain the observed benefits. Together, these findings indicate that acute nitrate ingestion enhances the ability to sustain high-intensity power output during repeated sprints, however, the underlying mechanism does not appear to involve measurable changes in central motor drive or microvascular responsiveness.

The results of our study regarding repeated Wingate tests align with previous studies suggesting dietary NO_3_^−^ can improve power maintenance and reduce fatigue during repeated sprints or intermittent bouts after supplementation. For example, the study performed by Esen et al. showed enhanced performance in intermittent running tests (Yo-Yo recovery test) in recreational athletes following dietary NO_3_^−^ supplementation [13], supporting its efficacy in anaerobic endurance efforts. However, it should be noted that not all studies pointed in the same direction. Martin et al. reported no enhancement of repeated sprint ability following NO_3_^−^ supplementation [14]. Another study by Jonvik et al. also reported no changes in peak power or mean power output in repeated Wingate tests across different levels of athletes with BJ, despite the evident increase in plasma nitrate and nitrite concentrations [15]. It’s unclear why such discrepancies exist, but they could possibly be attributed to differences in supplementation strategies (acute vs. repeated over multiple days), sprint protocols, as well as the recovery intervals, though clearly more research is needed for further clarification. Taken together, our findings reinforce the concept of dietary nitrate as an ergogenic agent for repeated anaerobic performance. However, the benefits could be protocol-specific, which depends on many factors and needs to be systematically explored.

Importantly, this study also examined the potential sex-dependent responses to nitrate. Females remain underrepresented in sport science research, and prior research on potential sex differences in response to nitrate has been extremely limited, and the results have been mixed. For instance, the study by de Zevallos et al. demonstrated improvements in exercise economy and endurance capacity in males but not in females following NO_3_^−^ supplementation [23]. In contrast, another study indicates that BJ does not significantly affect knee extensor maximal power and endurance in either sex [36]. Both studies, however, noted that such data on females are scarce. Physiologically, the estrogen-mediated NO synthase activity and vascular function [22] as well as the sex-dependent differences in muscular kinetics and composition [37], may alter the efficacy of NO_3_^−^ supplementation. Yet, in our study, both males and females showed robust and comparable improvements in repeated-sprint performance with BJ compared with PL, which indicates sex-based physiological differences do not significantly impact the acute ergogenic potential of nitrate in this context.

To explore the potential mechanisms, we also measured corticospinal excitability and microvascular function with dietary nitrate supplementation compared to PL. Given the previously reported greater blood flow to certain cerebral regions [19], it’s expected that nitrate intake might enhance motor cortex excitability or neural drive, thereby contributing to performance maintenance. However, the lack of changes in resting TMS parameters suggests no acute increase in corticospinal output, which implies that performance benefits for nitrate may predominantly be peripheral rather than central in origin. Additionally, we found no improvement in muscle microvascular reactivity at rest and no difference in muscle oxygenation trends during exercise with nitrate.

Combined with the lack of between-sexes findings in performance, it’s reasonable to assume that the specific mechanisms may involve non-estrogen-dependent peripheral pathways. One possibility is the modulation of muscle contractile function through the nitrate’s effects on increasing calcium handling kinetics [4]. Therefore, the force generation capacity is enhanced without either altering central drive or microvascular blood flow. However, such inference needs to be confirmed by further mechanistic studies. Additionally, NO_3_^−^ has also been shown to increase mitochondrial efficiency [3], which may facilitate ATP and phosphocreatine resynthesis during the passive recovery phases between sprints. Taken together, the observed ergogenic effects on repeated-sprint performance in the current study appear to stem from intrinsic muscle metabolic and contractile enhancements rather than central neural or vascular mechanisms, though further mechanistic research is warranted to confirm such an inference.

From a practical standpoint, our findings suggest that nitrate-rich supplementation can serve as a practical, accessible, and low-cost nutritional strategy. The energy requirements for the exercise modality we employed were similar to those of many team sports players who need to make repeated sprints during the course of a game, such as soccer, basketball, and rugby. Both male and female athletes can incorporate nitrate-rich foods or supplements into their pregame routine, and the absence of sex differences could imply the reduced need for sex-specific supplementation protocols. Given the established safety, affordability, and dietary nature of BJ, it is a practical option for recreationally active individuals seeking to enhance high-intensity performance.

Several methodological considerations should be taken into account when interpreting our study results. This study employed a resistance load of 7.5% of body mass across both sexes. While this is a common approach for studies involving both sexes to ensure comparability across performance indices, it should be noted that this load level may not be sufficient to elicit the true peak performance for males [38]. The choice of load level is unlikely to significantly affect the study results, as the main analyses focused on the change in performance across sprints within individuals and the interaction between supplement condition and sex. Nevertheless, future studies may consider sex-specific resistance loads to confirm that the present findings are robust across different loading conditions. Another aspect worth noticing is that due to technical limitations, the assessment of corticospinal excitability was done on the hand rather than the leg muscle, which could not lead to the complete elimination of corticospinal involvement during lower-limb maximal exercise. However, TMS test on the hand at rest could still be meaningful, as this choice reflects a feasible and validated approach in the field, even for studies primarily involving lower extremity muscles [39, 40]. In contrast, leg TMS is technically challenging and less reliable [41]. Additionally, the NIRS test was done at rest to reliably and correctly reflect microvascular function. However, this choice may limit the ability to capture the exercise-induced adaptations. In order to capture the potential changes in dynamic oxygen exchange, leg muscle oxygen changes were also measured during repeated sprint exercise and similar null results were detected. Ultrasound-assessed blood flow is an alternative method that can provide information regarding dynamic perfusion and could be applied in future similar studies to provide further mechanistic insights.

Limitations of this study must be addressed. First, participants of this study were young, healthy recreational athletes. Therefore, the study results may not be generalized to other populations, such as elite athletes or older athletes. Future research should examine whether training status or age alters the ergogenic effects of acute nitrate supplementation for repeated anaerobic performance. Second, this study employed an acute supplementation protocol. Whether shortterm or chronic supplementation would produce different results warrants further investigation. Third, the mechanistic measures in this study were all performed at rest. Given the null results, it’s reasonable to assume that measuring dynamic changes during exercise, such as motor unit recruitment patterns and phosphocreatine recovery kinetics could provide more insights not captured by the resting measures. Fourth, performance variables were expressed as percent changes from sprint 1 to sprint 4,. This approach, while practical and commonly used, results in a loss of temporal resolution for the performance variables, and larger studies with sufficient power to detect temporal changes should be conducted to understand the time course of dietary nitrate-elicited performance changes. Additionally, although the dose, testing timing (2.5 h after ingestion) and the use of commercially available supplementation were consistent with literature, the lack of biochemical analysis for plasma nitrate/nitrite levels limits confirmation of supplementation efficacy and interindividual responsiveness. Last but not least, our sample size was powered for the primary performance outcomes, and may have been underpowered to detect the sex by supplement interactions for exploratory mechanistic outcomes like TMS and NIRS tests.

## CONCLUSIONS

In conclusion, the results of this study indicate that acute dietary nitrate supplementation via BJ ingestion effectively mitigates fatigue development during repeated anaerobic efforts; yet, no sex differences were detected. The observed performance benefits occurred without detectable changes in the resting measures of corticospinal excitability or microvascular reactivity assessed in the present study. However, these null mechanistic findings should be interpreted with caution due to the technical restraints of assessment methods, and do not exclude exercise-dependent or muscle-specific neural and vascular mechanisms.
